# Hepatitis C Virus in Arab World: A State of Concern

**DOI:** 10.1100/2012/719494

**Published:** 2012-05-02

**Authors:** Mohamed A. Daw, Aghnaya A. Dau

**Affiliations:** ^1^Department of Medical Microbiology & Immunology, Tripoli Medical Centre, Faculty of Medicine Tripoli, P.O. Box 82668, Tripoli, Libya; ^2^Department of Vascular Surgery, Tripoli Medical Centre, Faculty of Medicine Tripoli, P.O. Box 82664, Tripoli, Libya

## Abstract

Hepatitis C virus has been considered to be one of the most important devastating causes of chronic hepatitis, cirrhosis, and hepatic cellular carcinoma. The prevalence of such virus varies greatly over the world. Arab world has a unique geography and consists over nineteen countries who share the same heritage and customs and do speak the same language. In this area, the epidemiology of hepatitis C is not well understandable. Hepatitis C virus was found to be endemic in Arabia. The serostatus of such virus was found to be variable among these countries with uniform patterns of genotypes. Such prevalence varies tremendously according to the risk factors involved. Blood and blood products, haemodialysis, intravenous, and percutaneous drug users, and occupational, habitual, and social behavior were found to be the important factors involved. Hepatitis C will have major social, economic, and even political burdens on such young and dynamic societies. Thus, strategies and clear policy of intervention are urgently needed to combat the consequences of HCV both regionally and at state level of each country.

## 1. Introduction

Hepatitis C virus has been considered to be one of the most potential pathogens that have hindered the medical community all over the world. Indeed, since its discovery in 1989, hepatitis C virus (HCV) has been recognized as a major cause of chronic liver disease worldwide and due to the surpassing hepatitis B virus [1]. The data reported by WHO estimated that the prevalence of HCV infection is 2.2%, and more than one million new cases were reported annually. Furthermore, an estimated 27% of cirrhosis and 25% of hepato-cellular carcinomas (HCC) worldwide occur in HCV-infected people [2]. Such infection increases tremendously among the developing countries particularly at those categories that were considered to be at a potential risk of acquiring hepatitis C virus.

 Arabian compound states are composed of unique geography being the central and transcross of the world. It is composed of more than nineteen countries with more than 350 million; they all speak the same language and mostly they have the same heritage and customs. Indeed it is the whole site of east and south of the Mediterranean basin up to the Atlantic cost in the west and Persian gulf and Indian Ocean south east. The African region is composed of the whole north African countries which are composed of approximately 23% of Africa, though the Asian region composed most of the north west of Asia and composed around 10% of Asia. Such area has over 30% of the world oil revenue, and most of them enjoy great wealth and prosperous capital.

The global epidemiology of HCV varies greatly allover the world, and different studies have shown such variations. Hepatitis C virus was found to be endemic in certain countries, and different studies have confirmed such endemicity [3]. It is important to consider the shortcoming of regional studies are in Arab world. Most of the epidemiological studies carried out individually based upon seroprevalence of HCV among specific groups. These include blood donors, heath care workers, or patients undergoing haemodialysis. Such studies were not representative of the community, they were usually carried out by independent scientists. Even though, some countries lack such studies.

Nowadays, HCV is responsible for infecting over 20 million in Arab countries, and without immediate and effective intervention, that number is predicted to increase tremendously in the next two decades. The objectives of this paper were to analyze the status of HCV epidemiology among the Arab countries and its variation accordingly and to highlight the future consequences and how to overcome the implications of such infection.

## 2. Epidemiology of Hepatitis C Virus in Arab World

The Arab world consists of heterogeneous countries with various levels of developments and different approaches of concepts and priorities. Infrastructure of health services is variable among these countries; some of them succeeded in adapting the modern routes of infection prevention; others fail and rarely adapt such recommendations [4]. However, most of them share the concept of bureaucracy and hierarchal among their health services and education [4, 5]. This will make the implementation of international standards indeed difficult to follow or adapt by their institution and health services personnel. HCV infection is a nagging issue in all the Arab countries; the estimated number of infected individuals in the Arab world by such virus reached 25 million with an average prevalence rate of 3.5%.  Table 1 shows the universal epidemiology of HCV among general population and patients undergoing haemodialysis and genotypes reported in each country. Such prevalence varies greatly from one region to another and even among the countries of the same region.

### 2.1. Hepatitis C Virus in Arabian Peninsula Region

 Arabian peninsula is unique geographical area a composed 35% of Arab world that it is composed of Saudi Arabia, Yemen, Oman, Qatar, Bahrain, UAE, and Kuwait. Saudi Arabia is the largest country not only in the Arabian peninsula but in the Arab world, and considered to be the richest. Hepatitis C virus is well studied in Saudi Arabia, and its serostatus is known among the different population [6]. The universal prevalence of HCV in the general population ranged from 1.1 to 1.7% [7]. Such prevalence was found to be high among the risk groups such as haemodialysis patients where it ranged from 18.6 to 56%, 40% among the haemophilia patients, and 94.8% among IVDs. The common genotype isolates were genotype 4 followed by rare ones 1a, 1b, and 3a [8–10]. Yemen is the second largest heavily populated and the poorest country in Arabian peninsula; the prevalence of HCV in Yemenis was found to be 1.7% among healthy volunteers though it reached 2.7% among blood donors [11, 12]. Such prevalence reached up to 60% in haemodialysis patients [13]. In the less populated Gulf states, HCV was found to be 0.9 to 1.5% in blood donors of Oman and 26% in haemodialysis patients [14], though it was found to be 40% among haemolytic Bahraini patients with HCV genotypes 4, and 1a and 1b as the commonly prevalent genotypes [15]. In Qatar, the prevalence rate among the general population reached 6.3% and 44.6% in dialysis patients [16–18], though in UAE, the prevalence was found to be 23% among haemodialysis patients, and the predominantly genotypes were 4, 3, and 1 [19]. In Kuwait, HCV is better known, and different studies were carried out to determine the prevalence of HCV as it was found to be 0.8% among the general populations and 71% among haemodialysis patients [20, 21]. The commonest genotypes were genotype 4 followed by genotype 1 [22]. However, it is clear that in the less populated states of Gulf area, detailed studies on HCV are lacking, and the magnitude of such problem is not well documented. Furthermore, the impact of foreign workers on the prevalence of HCV is not known, particularly as most of these workers are coming from regions that are considered to be endemic of HCV. Therefore, it is evident that the prevalence of HCV in Arabian peninsula ranges from to 0.8% to 2.1% among these countries apart from Qatar which reached 6% and that is difficult to explain (such data has to be taken with caution). Over one and half million were infected with HCV as HCV genotype 4 is more prevalent. This makes such region classified within an intermediate range worldwide as shown in Table 2.

### 2.2. Hepatitis C Virus in Sham Region

 This region is composed of Syria, Iraq, Lebanon, Jordan, and the existing Gaza Strip (ancient Palestine). Different studies were carried out in Syria on the prevalence of HCV among different categories [23]. The prevalence of HCV among Syrian was found to be 1% among the general population, though its remarkably high in the risk groups. Among haemodialysis patients it ranged from 24.4 to 88.6%, though it is 3% among health care workers and 60.5% among IDUs, and 1.96% among prostitutes [24]. The commonly isolated genotypes among Syrian people were genotype 4/1b followed by rare ones as 1a, 3a, and unusual genotype 5 was also reported [25].

 In Iraq, the prevalence of HCV ranged from 3.21 to 7.1% among the pregnant women and general population of four HCV genotypes, 1a, 1b, and 3a [26, 27]. A study carried out on Iraqi refuges showed further discrepancy in this regard; Chironna et al.; using HCV-RNA analysis found that the prevalence among these refuges was 0.1%, and HCV genotype 4c, d was the commonest [28]. Hence then further studies were needed to clarify the Iraqi serostatus. However, such prevalence was remarkably increased among haemophilia patients as it reached 66.0% [29].

 HCV is less frequent among Lebanese; it ranged from 0.16 to 1.22% among healthy volunteers, blood donors, and health care workers, though it was 27% among haemodialysis patients [30]. Genotype 4 was the commonest isolated genotype among the various groups studied followed by genotypes 1a and 1b [31].

 The collected data form Jordanian studies was found to be variable and even contradicting. The overall prevalence of HCV among Jordanian people varied from 0.65 to 6.25% depending on the population studied [32]. It was found to be 28% among haemodialysis patients and 40.5% in hemophiliac patients. The predominant genotype was found to be genotype 1a followed by 1b and 4 [33]. However, few studies were coming out from Gaza Strip concerning the prevalence of HCV where it was found to be 2.2%, though the most prevalent genotype was found to be genotype 4 followed by 1 and 3a [34, 35].

 Hence then, over two million with 0.7–6% of prevalence were infected in Sham region; the lowest prevalence was reported in Lebanon followed by Syria, Gaza strip, and then Jordan and Iraq, though the HCV genotype 4 was the commonest among all the regions a part from Jordan which do need further elucidation.

### 2.3. Hepatitis C Virus in Arabian Nile River Region

 Sudan and Egypt are the main countries to be considered in this region as they were resting on the Nile River which originated from the Ethiopian mountains to cross Sudan and pass via Egypt to the Mediterranean basin and indeed its one of the oldest ancient civilizations in the world. Hepatitis C virus has been intensively studied in Egypt, and great efforts have been shown in this important area [36]. The prevalence of such virus is reported to be higher in Egypt than in any other country. Indeed, it is a phenomenon that attracted the attention of many researches and scientists not only in Egypt but allover the world [37]. Despite the different speculation which has been postulated, the argument is still going on, and no obvious reason was proofed. The prevalence of HCV among the general populations was variable according to the studies conducted, as it varied from 13 to 22% accordingly [38, 39]. The prevalence of HCV among various groups and categories was intensively studied. It was found to be 14.5% among blood donors, 7.7% among health care workers, 12.1% of rural primary school children, 18.1% of rural villages residents, and 22.1% among army recruitments. It was even higher among risk groups, 70.4% in haemodialysis patients, 54.9% in multitransfused children, and 47.2% in chronic liver disease patients [40, 41]. The predominant HCV genotype among Egyptians was found to be genotype 4, particularly subtype 4a [42]. However, recent studies revealed that other genotypes and subtypes as 1a, 1b, 2a are also present indicating that HCV genotypes are extremely variable [43].

The status of HCV among Sudanese is also obvious as it is reported to be (2.2–3%) among the general population [44, 45]. High-risk population showed a higher prevalence of HCV, and it was found to be 23.7% in haemodialysis patients [46]. The major genotype isolated was genotype 4 with subtypes 4e, 4c, and 4d [47]. This was found to be similar to those genotypes isolated from Egypt.

Hence then, the Nile River region was considered to be the highest endemic area in prevalence of HCV with 15 million infected persons particularly genotype 4 as shown in Table 2. Further studies are needed to explore new methods for prevention and treatment.

### 2.4. Hepatitis C Virus among North African (Maghreb) Region

 The Arabian North African countries include Libya,Tunis, Algeria, Morocco, and Mauritania, (Mauretania). The prevalence of HCV and its genotypes among different populations at Maghreb countries is variable. In Libya, HCV has been intensively studied by Daw and his collaborators over the last ten years; the prevalence of HCV among Libyans was 1.2% though among hemodialysis was 20.2% [48]. Recently, a comprehensive study consisted of 1240 Libyan patients was carried out [49]. Different genotypes were reported in the study; genotype 4 was the commonest (35.7%), followed by genotype 1 (32.6%). According to subtypes, 28% were unclassified as genotype 4, 14.6% were genotype 1b, and some patients were infected with more than one subtype (2.3% genotype 4c/d, 1% genotype 2a/c). Genotype 1 was the commonest among males, while genotype 4 among females. Despite the obvious role of prevention and control of such virus and data available by Libyan researchers, the national authority failed to adapt these regulations at a national level, though every centre has its own way of adapting such recommendations.

Different studies were also carried out in Tunis [50, 51]; the prevalence of HCV reported among Tunisians was 0.4 to 0.7%; the lowest among was the Maghreb countries, though such prevalence was high in haemodialysis patients as it reached 51% [52]. The commonly isolated HCV genotypes among Tunisians were 1b which accounted for 79%, followed by genotypes 1a(5%), 2a(7%), 2b and 3a(3%), and 4a (1%), respectively [53]. In Algeria, a national seroprevalence study was carried out covering healthy blood donors and pregnant women. Prevalence of HCV among these two groups was 0.18 and 0.19%, respectively, though it was 2.5% among the general population [54–56]. However, further studies on genotype and analyzing the serostatus on other risk population as haemodialysis and haemophilia are needed.

In Morocco, the prevalence of HCV was reported to be high. It varies greatly from one study to another. It was found to be 1.93% in a study carried out by Beghdadli et al. [57], while it reached 7.7% in another study by Benouda et al. [58]. Such prevalence was high among haemodialysis as it reached 42.4%, though it was 35.1% within the haemophiliacs patients [59]. The most prevalent genotypes reported among the Moroccans are 1b (47.6%), 2a/2c (37.1%), and 1a (2.8%) [60]. Furthermore, the prevalence of such genotypes varies according to age; genotype 1b is more prevalent among older patients, whereas subtype 2a/2c is mainly found among younger ones. 

Despite the clear picture of HCV epidemiology in Libya, Tunis, and to a certain instant in Algeria and Morocco, such data is lacking in Mauritania; anti-HCV was detected in 1.1% among blood donors [61]. It has been assumed it will be the same pattern as the rest of other Maghreb countries though no reported studies in this subject, and hence then, further studies are needed to clarify such assumption.

The epidemiology HCV among the Maghreb countries was found to be variable, and around one million and half people were infected. It is less prevalence among the Tunisian population, followed by Libya with low prevalence, then Algeria and Mauritania which are classified as moderate and later Morocco with a higher prevalence; furthermore HCV genotype 4 is the predominant one among Libyans, though genotype 1 is more common in the rest of Maghreb countries. However, it was difficult to explain such variation as the people in these countries are very interacted and sometimes difficult to differentiate between. This may be related to the nature of the studies we referred to, and hence then, further detailed studies are needed.

## 3. Factors Associated with Transmission of Hepatitis C Virus in Arabia

 The epidemiology of HCV among Arabs is unique in its nature, and varieties of risk factors have been found to be associated with its high prevalence rate. However, the emphasis on these factors may vary from one country to another as shown in Table 3.

### 3.1. Blood and Blood Products

 HCV has been known to be the most common blood-borne virus over the world particularly among the developing countries. Blood and blood products remain a major cause of spread of HCV among Arab countries, and most of them fail to fulfill the criterions of modern blood transfusion system. Different studies have shown the impact of blood transfusion on the status of HCV in these countries. Studies from Jordan showed that HCV infection was detected in 34% of patients who had received blood transfusion, compared to only 16% of those who received no transfusion [62]. The picture in Syria is also damming where 50.4% of HCV-dialyzed patients have previous history of blood transfusion. In Yemen, 80% received blood transfusion were infected with HCV compared with 20% who received no blood [63]. In Libya, a history of blood transfusion was reported in 22.7% of those with HCV [49]. HCV was found to be remarkably high among thalassemia patients who received repeated blood transfusion; it was found to be 67.3% in Iraq, 42.4% in Morocco, 40.7% in Jordan, and 40% in Saudi Arabia [64].

 In Arab countries, blood transfusion is still a problem due to the lack of organized infrastructure and highly trained and qualified staff. The main sources, of blood donation are usually relatives and friends of the patients who come on social pressure and due to the fear of the death of a patient (i.e. relative). Donors usually come in emergency time, and questions about high-risk behaviors are seldom asked [65]. Therefore, resources and organization should be available to recruit altruistic volunteers.

The current data indicate that all the Arab countries undertake anti-HCV screening mandatory in all blood banks, though in some countries, patients may have to pay for such tests. The main concern is the reliability of such tests, and some data indicated that antibody tests for HCV genotypes/subtypes common in Europe and North America are not well applicable on the sera tested from developing countries though most of Arab countries import such tests [66]. Furthermore, rapid tests and some lots of ELISA failed to detect HCV-reactive sera and up to 1% of seronegative blood units tests positive for HCV-RNA using RT-PCR technology [67]. Therefore, even when HCV screening test is performed the risk of becoming infected is still visible. This however makes it necessary to add new molecular tests such as the determination of HCV RNA by nucleic acid technology (HCV-NAT) to the already performed tests in blood donation which should be coupled with meticulous professional assessment to the effectiveness of such tests [68].

 The recent uprising in Arab countries which may brought inspiration to Arab citizens left some of health care services in a chaotic situation. In Libya, such crises left an estimated 50.000 killed and over 120.000 injured; screening of blood in war field was mainly limited quick screening tests and sometimes only matching blood groups to save lives [69]. This will have great impact on the prevalence of HCV and the consequence among the infected victims in such country.

 Blood transfusion and hospital-associated practice were found to be important risk factors for HCV among Arabian countries. These conditions can be overcome by development of a fair and organized system of blood screening and transfusion. National and international standards should be implemented, and firm revision for such standards and its implantation should be regularly revised. Further, independent or even legal bodies should observe that such implantation and legal action should be taken.

### 3.2. Haemodialysis Practice

 The prevalence of HCV among haemodialysis patients varies greatly from one region to another, and it has been reduced drastically over the years in developed countries. In Japan and UK, it was found to be (1.2%) and (4%), respectively, though in certain African countries, it reached up to 80% as in Senegal [70]. Among Arab countries the picture of serostatus of HCV among HD patient is peculiar. Such prevalence is higher than that in the general population, blood donors, and even among those with other risk factors. Furthermore, it is more complex and varies greatly from one country to another and even among different dialysis centres in the same country [71]. Indeed, the prevalence of HCV among the haemodialysis patients in any Arabic state per se surpasses any other international centres in the developed countries, who follow the strict regulations of HCV prevention in such setting. The prevalence of HCV varies from 20.2% to 76% in such countries, the highest prevalence was reported in Morocco (76%), Kuwait (71%), and Yemen (61%), though the lowest was reported in Libya (21%) as shown in Table 1.

In Arab countries, blood transfusion has played an important role in transmission of HCV in HD patients. Furthermore, dialysis centres are usually overloaded with patients, and there is a shortage of material support (filters), which leads to multiple use of the filter without proper sterilization in some cases. In addition, there is an insufficient number of dialysis machines; the number of patients dialyzed per machine is twice that in developed countries. Clusters of HCV infection have been noticed in many haemodialysis centres as in Syria, Sudan, Morocco, Yemen, Kuwait, and Bahrain which may suggest large outbreaks in such centres due to patient-to-patient transmission via HCWs' hands [72]. Further evidence from CDC's investigators raises one possible way in which inappropriate use of medication vials intended for single person may result in large HCV outbreaks in haemodialysis units. This is however to be confirmed by phylogenetic analysis of subgenomic regions of HCV [73].

Hepatitis C virus in plasma remains viable and detectable after drying and environmental exposure to room temperature for at least 16 hours; therefore, blood-contaminated surfaces and objects can serve as sources for HCV transmission. Generally, such transmission among haemodialysis units is considered to be nosocomial with possible factors being failure to disinfect devices between patients; sharing of a single-use vials of infusions; poor sterile technique; poor cleaning of dialysis machines; poor distance between chairs in addition to dialyser reuse, duration, and frequency of dialysis [74]. HCV can hardly be transmitted during dialysis procedures when state-of-the-art machines are used, where most of these countries offered to have. The prevention of HCV among HD is visible and such risk could be drastically reduced. Erythropoietin should be used to reduce the need of blood in HD patients, and proper nosocomial prevention program should be implemented. This could be advanced and supplemented by training, proper staffing and a sufficient supply of materials and disposables according to the actual need.

### 3.3. Intravenous Drug Users (IDUs) and Prisoners

 Transmission of HCV was strongly associated with intravenous and percutaneous drug users (IDUs). The hepatitis C European network for cooperative research group reported a prevalence of HC of 80% among intravenous drug users (IVDU), 40% in Thailand and up to 74% in Australia [75]. In injecting drug users (IDUs) population there has been increased shifts among addicts from inhalatory to injectable drugs due decrease in quality and availability of heroin. Further, the effect of injecting drugs is more intense and satisfying as HCV is found in a high concentration in spoons and rinsing liquids that could be used in association with needle drug use.

Injecting illegal drugs, sex behavior, and imprisonment are considered to be shameful and unrespectable misconduct among Arabian society. Individuals usually hide and deny such act even if they did it, a state to be taken with caution when taking history of risk factors particularly among blood donors. More than a third of all prisoners and more than 80% of injecting drug users, were positive for antibodies to hepatitis C virus [76]. Alcohol consumption was also found to accelerate the course of chronic hepatitis C. In Arab countries, alcohol is licensed for sale except in Libya and Saudi Arabia. Such important risk factors rarely are covered by Arab researchers; very little data exist regarding the prevalence of injection drug use combined with Alcoholism and imprisonment and its contribution to HCV infection in the Arab world [77]. In Lebanon HCV among IDU was found to be 52.8%, with no difference in marital status, and in prisoners, it was found to be (3.4%); tattooing was also associated with HCV [78]. In Syria, the prevalence of HCV among Syrian prisoners was found to be 60.5 among IDUs and 1.96 among prostitutes [79]. A study in Saudi Arabia showed that intravenous drug addicts have 14% of exposure rate [80]; another study in Libya found that HCV was 15% among IDUs [49].

 Such behaviors are significant factors for HCV infection, and this may become one of the prison system major health concerns over the next two decades. Suggesting that measures to minimize the spread of hepatitis within these groups are essential and education programs seem to be most appropriate way to minimize such risk [81]. This emphasizes that the time has come for national and regional policy makers, researchers, and clinicians among Arab countries to take prisons as important health risks and never to be considered as disadvantaged population.

### 3.4. Occupational Transmission of HCV

 Hepatitis C virus has been known to be an important nosocomial pathogen, and several outbreaks have been linked to breaches in standard precautions for blood-borne infections during nursing procedures or interventions such as colonoscopy surgery and dialysis [82]. In Arab countries where HCV infection is endemic in the general population, hospitalization and invasive procedures do appear to be significant risk factors for HCV infection. Different studies have shown that the prevalence of HCV among HCW was reported to be 3% in Syria, 0.4% in Lebanon. In Egypt, health-care-related risk factors contributed immensely in HCV infection particularly those aged over 20 years old. Surgical practice such as suture was associated with (32.3%), IV catheters (11.7%), though urinary catheter (5.2%), and it was much higher in dental practice as it reached (62.2%) [83].

The occupational transmission was better studied and the picture was more obvious in the North African region. In Libya, the commonest predisposing factor reported was mainly medical-related transmission of HCV, such as hospitalization and/or surgical procedure risk which accounted for 33%, and history of dental procedure for 15.5%, when compared with other risk factors [69]. The prevalence of HCV among Libyan children has an immense increase in 1998, where 420 children have found to be coinfected by both HCV and HIV in a short period of time with no evidence of vertical transmission [84]; this unusual increase in the prevalence of HCV among Libyan children was associated with Bulgarian nurses saga. However, such scrutiny is still under a major scientific dispute among scientists [85].

 In Tunis, 51% of HCV cases were reported to be nosocomial rather than transfusion related. In Algeria, occupational exposure seems to be an important risk factor for HCV transmission [54]. Needle stick injuries represented 81% among the health care personnel in Algerian hospitals mainly due the mismanagement of health care waste produced in the hospital environment [57]. Hence then, strict adherence to the international standard precaution should be adapted on a national level allover Algeria.

The assessment of sharps use in Arab countries indicates that injections sometimes were given in a way that may harm the patient. Determinants of these unsafe injection practices include the lack of single-use injecting devices, the lack of awareness of the risk of HCV and HIV infection associated with unsafe injections, and the absence of sharps waste management [86]. Infection control practices in health care settings needs to be reviewed and improved to prevent nosocomial and iatrogenic transmission of HCV (and other blood-borne pathogens). All health care workers should regard patients as infected with a transmissible blood-borne agent.

### 3.5. Habitual and Community-Associated Factors

 Community has been considered to be an important source for hepatitis C, and up to 50% of individuals deny exposure to any of these known risk factors where, infection is often designated as community acquired. Different habitual and biosocial behaviors have reflected on the endemicity of HCV among Arab countries. Indeed children and young adults in Arab countries have a relatively high HCV prevalence, although it is less than in the older population, which suggests that HCV transmission continues in these communities [87]. A variety of contributing factors may be involved, and it becomes imminent that strategies should be implemented to overcome such potential risks. Unsafe use of unsterilized injection is a major risk factor of HCV in Arab communities. It is likely that injections given in rural communities by both traditional and nontraditional health care providers are an important cause of HCV transmission, particularly in countries like Egypt, Sudan, Mauritania, Morocco, Algeria, Iraq, and Yemen. The importance of combating this ubiquitous risk in prevention programs cannot be overstated. A study carried out by World Health Organization (WHO) on the global use of injection has shown that most of the Arab countries have serious problems in using injection in their communities. Eastern Mediterranean region D which mainly includes Egypt, Iraq, Morocco, Yemen, and Sudan has the highest injection per person associated with lack of sterilization. Interestingly, injection practices are safer in sub-Saharan African than in these countries [88]. The same study have also shown that African region which included among the states studied Algeria and Mauritania has also shown a high incidence of unsafe use of injection both health care settings and among community [88]. Furthermore, there is an ongoing speculation that the use of parenteral antischistosomal therapy campaigns contributed to the establishment of a large reservoir of HCV infection in Nile River region [89].

Habitual and social behaviors have been found to influence HCV infection in Arab countries. Circumcision, one of the potential culturally influenced exposures to HCV in the community, occurred too frequently. Males over the age of 20 had been circumcised either by informal health care providers or by community physicians or nurses who were more likely to be infected with HCV than those circumcised by surgeons [83, 90]. Interestingly, marriage was found to be another strong risk for HCV. In UAE, increased prevalence of HCV among spouses was detected with longer duration of marriage being an important risk factor, such infection was passed to children at an early stage [91]. A major logistic-regression model study carried out in Egypt found that the association of HCV in spouses could be the result of sexual transmission or common exposures. Furthermore, they have reported that spouses of patients with HCV have an increased risk for acquiring HCV, and this risk increases with age and is proportional to the duration of marriage [92]. However, unique community-acquired exposures such as smoking goza pipes, shaving by a community barber, ears piercing which common practice among Arab communities were never reported as a cause HCV infections [83, 93]. Elucidating the relative contribution of various modes of transmission of blood-borne viruses could help to direct our efforts towards appropriate intervention strategies in order to achieve viral hepatitis prevention and control. Community centres and counseling institutions should be established in order to understand and facilitate to overcome such existing risk factors. This will help persons in need by modifying behaviors and highlighting risk factors in the setting in which the patient is identified and could be referred to such a convenient community centers.

## 4. Strategies and Preventive Measures to Control HCV Infection in Arab Countries

Indeed, the status of HCV among Arabic countries should be a worrying issue to all sectors involved in public health, particularly those who are in close contact with patients and strategists who should plan for new healthy look of the Arabic societies [5], despite that there is clear difference among the Arab countries regarding the prevalence and the problematic issues of HCV, which should be eventually taken in consideration regarding the prevention of HCV at each country. Major measures should be implemented as illustrated in Table 4. These include immediate primary intervention (short-run) strategies and long-run prevention strategies. Such prevention strategies should target reduction of transmission of HCV, particularly among those at risk of acquiring the virus. Risk-reduction counseling and HCV screening program were directed to specific population as suggested by Centre for Disease Control (CDC) which may include persons at long-term dialysis, chronic liver diseases patients, health care workers after needle stick injury, and children of HCV-positive mothers [94]. Preventive measures should be directed towards individuals or populations at specific settings such IDUs/STD and prisoners. Hospitals and health care centers should adapt universal and specific infection control programs targeting not only the status of nosocomial infection per se but also particularly those units or persons who were more prone to HCV infection.

 Specific prevention programs should take place by adapting better cleansing and standard sterilization methods to stop nosocomial and iatrogenic transmission of HCV. Patient-care practices associated with higher HCV prevalence among chronic hemodialysis units should be identified, and recommendations and precautions in these settings should be adapted. This includes routinely wearing gloves; restricting the use of common supplies, instruments, and medications for multiple patients; prohibiting the use of mobile carts within treatment areas to store or distribute medications and clean supplies [95, 96].

 Further programs should include obligatory advanced laboratory screening methods for blood and blood products and reduction of number of transfusion-related transmissions and promote judicious injection and parenteral medications among doctors and patients. Once patients are found to have hepatitis C, they need to be counseled and clinically evaluated, to reduce the risk of transmission and stop the progression of the disease, respectively. Patients with chronic HCV are susceptible to HAV, or HBV infections should be vaccinated particularly among the Arab countries as the prevalence of these two viruses is high [97]. Further HCV is considered to be an opportunistic disease in persons with HIV infection [98]. Hence then, the safety and efficacy of antiviral therapy INF-& and/or INF-& plus ribavirin for HCV coinfected patients must be evaluated scientifically through clinic trial in Arab world [99]. The implementation of new antiviral treatment and the role of genotyping also have be assessed and appropriate strategies for management of end-stage liver diseases have to be rigorously investigated [100].

### 4.1. Implications for the Future Challenges

 Hepatitis C virus is a serious ongrowing problem in Arab countries, and it has great social-economic impacts which may touch the future of the young generation and influence the infrastructure of such dynamic states. Despite the great capital and natural resources that this nation has, none of these countries have shown meticulous and clear national or regional scientific plans to combat the future damage that HCV may cause. Prevalence of HCV is destined to increase further among Arab countries which should be an alarming issue for further measures. A recent forecast modeling study for prediction of hepatitis C seropositivity among Libyans has shown that in 2020 the HCV will increase by threefold [101]. Another modeled incidence study from Egypt showed that the prevalence of HCV is expected to be continuing at a rate of *≈*6.9/1,000 persons per year, indicative of possibly ongoing hyperepidemic transmission in this region [93]. This will have an immense effect on the Arab society with great social clinical and economic implications. Hence then, radical improvement in local infection control measures capable of limiting viral spread among general population should be implemented. Further prospective studies in various population groups are needed in most of the Arabia to generate reliable data on the clinical significance of HCV and its genotypes to hamper such rigorous implications.

## Figures and Tables

**Table 1:** Prevalence of hepatitis C virus and its common genotypes among Arab countries.

Region/country	Prevalence (%) of HCV among		Genotypes/subtype	
	Population [M]	Haemodialysis	More frequent	Less frequent
Arabian peninsula region				
Saudi Arabia [Sa]	23.513 (1.7)	55.7	4	1a 1b 3a
Yemen [Ye]	23,701 (2.1)	62.7	4	NA
Oman [Om]	3.2 (1.2)	26.5	4	NA
Bahrain [Bh]	0.656397 (1.7)	29.24	4	1a, 1b
Qatar [Qr]	0.793341 (6.3)	44.6	4	2, 3, 4
UAE	4,496 (2.3)	37	4	3a
Kuwait [Kt]	3.442 (0.8)	71.0	4	1

Sham region				
Iraq [Irq]	23 (3.2)	35.9	4	1a, 1b, 3a
Syria [Sy]	20.056 (1)	48.9	4	1b, 1a, 3a, 5*
Lebanon [Lb],	3.678 (0.7–1)	27	4	1a, 1b, 2, 3
Jordan [Jr]	5.307 (0.65–6.25)	34.6	1a	1b, 4
Gaza Strip	1.5 (2.2)	31.3	4	1, 3a

Nile river region				
Egypt [Eg]	80 (13–22)	40	4	1a 1b 2a
Sudan [Sud]	40.219 (3)	23.7	4	4e, 4c, 4d

North African region				
Libya [Ly]	6,037 (1.2)	20.5	4/1	2a, 2b, 2c, 3a
Tunis [Tu]	10.102 (0.4–0.7)	51	1b	2a 2c 1a
Algeria [Alg]	35.100 (1.8)	63	NA	NA
Morocco [Mo]	35.757 (7.7)	76	1b	2a 2c 1a
Mauritania [Mu]	3.365 (1.8)	68	NA	NA

NA: no data available, *less frequent type.

**Table 2:** Categorization of Arab countries according to the prevalence hepatitis C virus.

Category	Prevalence	Country
Low	1–1.9	Libya, Tunis, Oman, Saudi Arabia, Kuwait, Bahrain, Syria, Lebanon
Moderate	2.0–2.9	Algeria, Mauritania, Yemen, Gaza Strip
High	3–3.9	UAE, Iraq, Sudan
Very high	>4	Egypt, Morocco, Qatar, Jordan

**Table 3:** Factors associated with the Transmission of HCV among Arab countries.

Risk factor	Extent of Exposure among each country		
	Low	Moderate	High
Blood transfusion	[NO]	[All in this range]	[NO]
Haemodialysis	[NO]	[NO]	[All in this range]
Nosocomial transmission	[NO]	[NO]	[All in this range]
Health care workers	[NO]	[All in this range]	[NO]
Invasive Medical procedures	[NO]	[All in this range]	[NO]
Dentistry Practice	[NO]	[Lb, Ly, Tu, Sa, UAE, Om, Bh, Qr, Jr, Kt]	[Eg, Ye, Sud, Mo, Mu, Alg, Irq, Sy]
Laboratory services	[NO]	[Lb, Tu, Ly, Jr, Sy, Sa, Bh, Kt, Qr, Om, UAE]	[Mu, Mo, Alg, Sud, Irq, Ye, Eg]
Hospital Waste Handling	[NO]	[Lb, UAE, Sa, Ly, Lb, Bh, Qr, Om, Tu, Jr, Sy, Kt]	[Alg, Mo, Sud, Eg, Ye, Irq, Mu]
IVDA	[NO]	[NO]	[All in this range]
Habitual	[Ly, Tu, Lb, Sa, Jr, Sy]	[Alg, Irq, UAE, Qr, Bh, Kt, Om]	[Eg, Mr, Mo, Ye, Sud]
High risk behavior	[Ly, Sa, Ye, Sud, Mu, Om, Kt]	[Alg, Tu, Eg, Jr, Sy, Lb]	[Mo, Bh, Irq, UAE, Qr,]

Low: <5%, Moderate: 5–20%, High: > 20%, NO: No country in this category.

**Table 4:** Preventive and combat strategy programs for hepatitis C virus in Arab countries.

(1) Immediate continuous prevention strategies
(A)* Universal prevention planning *
(i) Well-planned educational programs regarding the risk of HCV both at the community and health institutions levels
(ii) Implementation of international and national guidelines regarding the prevention of HCV particularly at special hospital
settings as blood banks and haemodialysis units and high risk groups at the community
(iii) Strict adherence to such guidelines and regular assessment to its applications
(iv) Introducing specific patient-care practices
(B)* Special settings prevention programs *
(i) Blood and blood products, HCV screening program and using thioproprin, haemovigilance
(ii) Haemodialysis; strict adherence to nosocomial prevention program; review practices to ensure they are consistent with
recommendations and applied routinely,
(iii) Laboratory and health care; improving laboratory testing, better sterilization, safer injection, and less exposure to blood
products
(2) Long-run preventive strategies
(A)* Universal preventive planning *
(i) Vigilance and health alert programs which should report any problem and allow to interfere at any time
(ii) Elucidation is needed for better prevention, screening, and updating HCV treatment
(iii) Prevention of HCV infection progress
(iv) Eradicate the massive use of unsafe medical procedures
(B)* Special settings preventive planning *
(i) Injecting drug users
(ii) HIV-HCV coinfected patients
(iii) Prisoners inmates
(3) Research planning and priorities
Well-designed research programs should be established both at country level and regional levels which may include
(i) Population-based surveillance studies
(ii) Evaluation of safety and efficacy of antiviral therapy for HCV alone and with other coinfected viruses particularly HIV
(iii) Further evaluation of iatrogenic causes of HCV transmission
